# Sex-dependent differences in the gut microbiota following chronic nasal inflammation in adult mice

**DOI:** 10.1038/s41598-021-83896-5

**Published:** 2021-02-25

**Authors:** Yuko Mishima, Takako Osaki, Atsuyoshi Shimada, Shigeru Kamiya, Sanae Hasegawa-Ishii

**Affiliations:** 1 Department of Immunology, Faculty of Health Sciences, Kyorin University, 5-4-1 Shimorenjaku, Mitaka, Tokyo 181-8612 Japan; 2 Department of Infectious Diseases, Kyorin University School of Medicine, 6-20-2 Shinkawa, Mitaka, Tokyo 181-8611 Japan; 3 Pathology Research Team, Faculty of Health Sciences, Kyorin University, 5-4-1 Shimorenjaku, Mitaka, Tokyo 181-8612 Japan

**Keywords:** Immunology, Microbiology

## Abstract

A growing body of evidence suggests a relationship between olfactory dysfunction and the pathogenesis of mental disorders. Our previous studies indicated that chronic nasal inflammation caused loss of olfactory sensory neurons and gross atrophy of the olfactory bulb, which may lead to olfactory dysfunction. Simultaneously, increasing numbers of reports have elucidated the importance of gut microbiota to maintain brain function and that dysbiosis may be associated with neuropsychiatric disorders. Here we examined whether chronic nasal inflammation perturbed gut microbiota and whether there were sex differences in this pattern. Eight-week-old C57BL/6 mice repeatedly received bilateral nasal administration of lipopolysaccharide (LPS) 3 times/week to cause chronic nasal inflammation or saline as a control. At 9 weeks, cecal feces were used for 16S metagenomic analysis and whole blood and fresh tissue of spleen were used for ELISA analyses. Microbiome analysis demonstrated a remarkable change of the gut microbiota in male mice with chronic nasal inflammation which was different from that in female mice. In both mice, systemic inflammation did not occur. This has shown for the first time that chronic nasal inflammation correlates with sex-dependent changes in the gut microbiota. The detailed mechanism and potential alteration to brain functions await further studies.

## Introduction

Mental disorders include depression, bipolar disorder, schizophrenia and other psychoses, dementia, and developmental disorders including autism. The number of patients with a mental disorder is increasing and about 300,000,000 people experience depression in the world (WHO, 2018). This may correlate to the increase in the number of suicides associated with mental disorders, which is a worldwide problem. Given that many patients with mental disorders such as depression, autism, schizophrenia, and Alzheimer’s disease are reported to have impairment of sense of smell^1–3^ and that chronic rhinitis and rhinosinusitis are a risk factor for depression and anxiety^4,5^, the olfactory system is purported to be associated with the pathogenesis of mental disorders. However, the mechanisms connecting the nose to the brain are still an enigma.

Nasal inflammation may be a trigger for brain tissue damage and impairment of function. In our previous studies, we demonstrated that chronic nasal inflammation causes loss of olfactory sensory neurons (OSNs) and gross atrophy of the olfactory bulb (OB), the first relay station of the olfactory neurocircuit in the central nervous system. In the OB, glial cells were activated, pro-inflammatory cytokines were elevated, and subsequently tufted cell-related neurocircuits underwent degeneration in a mouse model with chronic nasal inflammation^6–8^. These results suggest that an olfactory neural pathway is a route through which nasal inflammation damages the brain. In fact, patients with mental disorders, as well as chronic rhinosinusitis, have smaller OB volume^9,10^.

Microbiota resides in various sites throughout the body, such as the nasal and oral cavities, lung, gut and even the brain, with the biggest colony within the gut^11^. The gut microbiota interacts with the host through the immune, metabolic and nervous systems and influences the brain function^12,13^. Therefore, an unbalanced composition of microbiota, termed dysbiosis, breaks down an individual’s homeostasis and is associated with a variety of disorders including inflammatory bowel disease, metabolic syndrome, obesity, and diabetes^14,15^. Recent reports indicate that neurodegenerative and neuropsychiatric diseases such as Alzheimer’s disease, Parkinson’s disease, multiple sclerosis, autism spectrum disorder and depression are also related to dysbiosis^16,17^, indicating the strong connection between the gut and the brain. Antibiotics are a trigger to perturb gut microbiota, which induces a loss of taxonomic and functional diversity combined with a reduced colonization resistance against invading pathogens^18^. The mode of delivery (cesarean or vaginal birth) and of feeding (breast milk or synthetic milk) can also influence the gut microbiota in the process of development in a newborn baby^19^. However, other factors perturbing adult gut microbiota are not well documented.

In the present study, we addressed the question of whether chronic nasal inflammation would cause perturbation of gut microbiota. We also compared the results between sexes.

## Results

### Nasal inflammation

After repeated administration of lipopolysaccharide (LPS) to bilateral nostrils, inflammatory cells such as macrophages (F4/80-positive), neutrophils (Ly-6G-positive), T cells (CD3e-positive) and B cells (CD45R-positive) locally infiltrated some regions of the olfactory mucosa (OM) in male and female mice, while these cells were not observed in the OM of saline-treated mice (Supplementary Figure S1). Some macrophages (CD11b-positive) produced interleukin (IL)-1β, a proinflammatory cytokine, in the LPS-treated OM in male and female mice (Supplementary Figure S1). During repeated administration of LPS, numbers of mature and immature OSNs (OMP- and GAP43-positive, respectively) gradually decreased similarly in male and female mice (Supplementary Figure S2).

### Gut microbiota

#### Diversity

**Figure 1:**
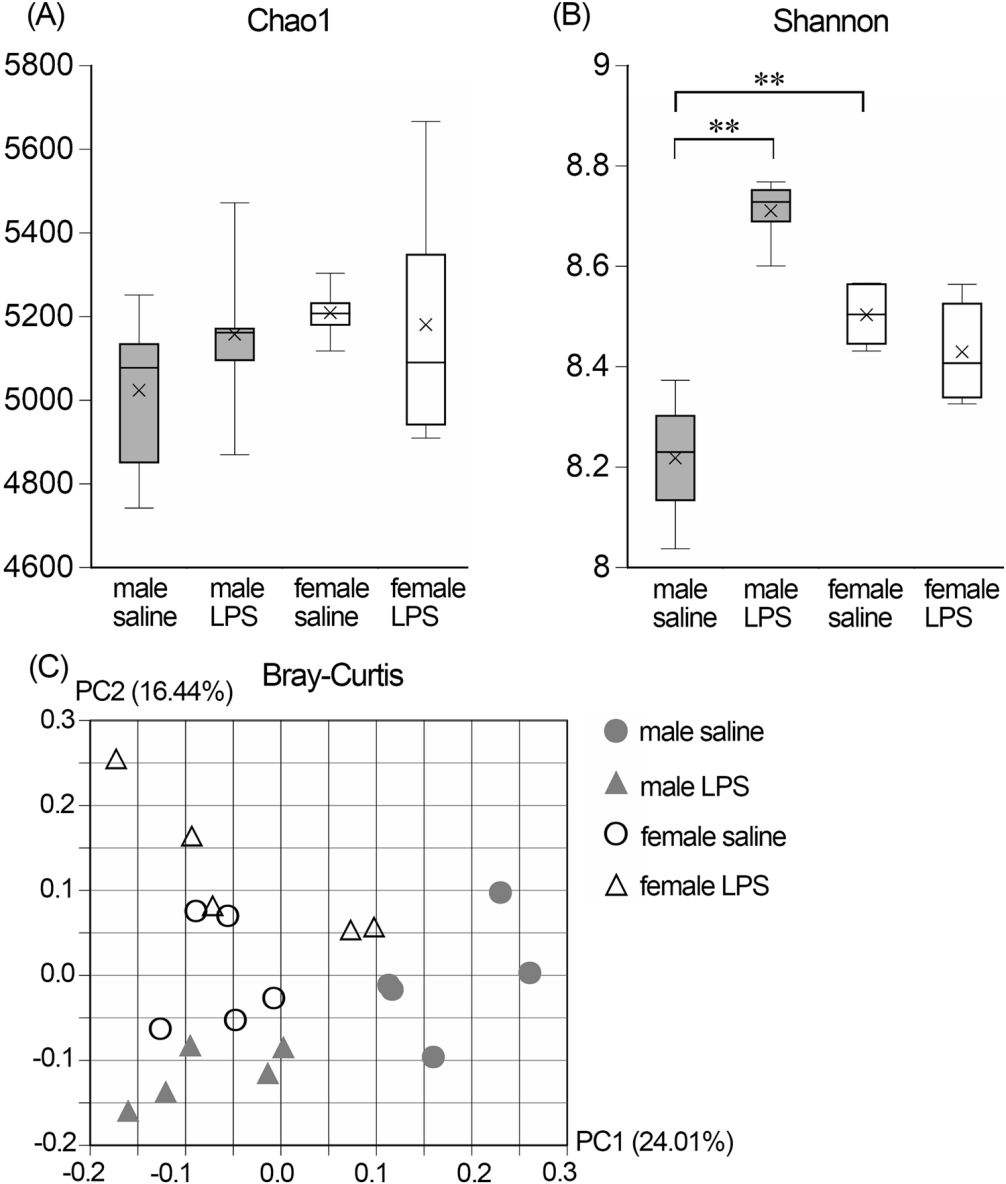
Diversity of gut microbiota. (**A**) Chao1 index. (**B**) Shannon index. The middle line in the box plot represents the median value, and the box is drawn indicating the 25 to 75% quartiles. Whiskers show minimum and maximum values and the ends of the whiskers represent the non-outlier range. Mean values are shown as ×. Data were analyzed with a 2-way ANOVA (sex × treatment) with Tukey’s HSD post hoc test. **p < 0.01. (**C**) Principle component analysis (PCA) based on Bray–Curtis dissimilarities among all sample sets. Dots with different symbols indicate samples collected from different groups. PC1: first principal coordinate, percent variation 24.01%; PC2: second principal coordinate, percent variation 16.44%. n = 5 for each group.

First, we examined Chao1 and Shannon indices to examine the α-diversity. As shown in Fig. 1A, the Chao1 index did not change in LPS-treated mice compared to saline-treated control in male or female mice. The value was also similar in male and female mice. In contrast, Shannon index significantly increased in the male LPS-treated compared to male saline-treated control mice. The value was significantly higher in female than in male saline-treated control mice (Fig. 1B).

Next, we performed Bray–Curtis PC (principle component) plots to compare the β-diversity amongst the 4 groups. The values of the male saline group distributed apart from those of the male LPS group, while values were more accumulated in female mice (Fig. 1C).

#### Phylum analysis

**Figure 2:**
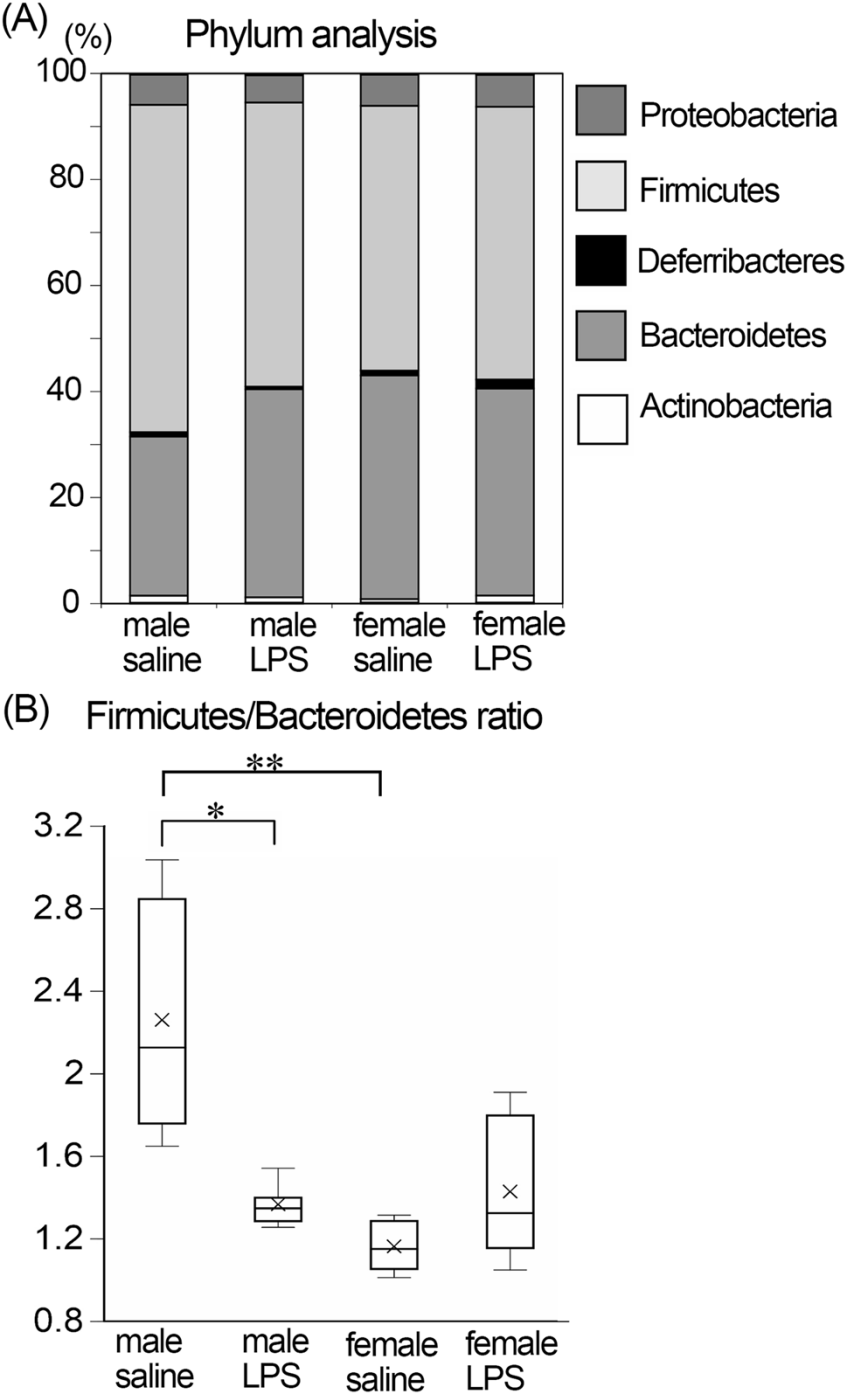
Phylum level analysis of gut microbiota. (**A**) The relative abundance of gut microbiota at phylum level. (**B**) The relative ratio of Firmicutes/Bacteroidetes in gut microbiota. The ratio significantly decreased in male LPS-treated compared to male saline-treated mice. *p < 0.05, **p < 0.01. n = 5 for each group.

At the phylum level of the taxonomy, Bacteroidetes and Firmicutes were the two predominant phyla in the gut and accounted for more than 90% of all bacteria in mice (Fig. 2A). The ratio of Bacteroidetes increased with a relative decrease in Firmicutes in male LPS-treated compared to male saline-treated mice, whereas the ratio did not change in female mice. The ratio of Firmicutes to Bacteroidetes significantly decreased in male LPS-treated compared to male saline-treated mice, while the value did not change in female mice (Fig. 2B). The ratio was significantly higher in male saline-treated compared to female saline-treated mice (Fig. 2B).

#### Family and genus analysis

**Figure 3:**
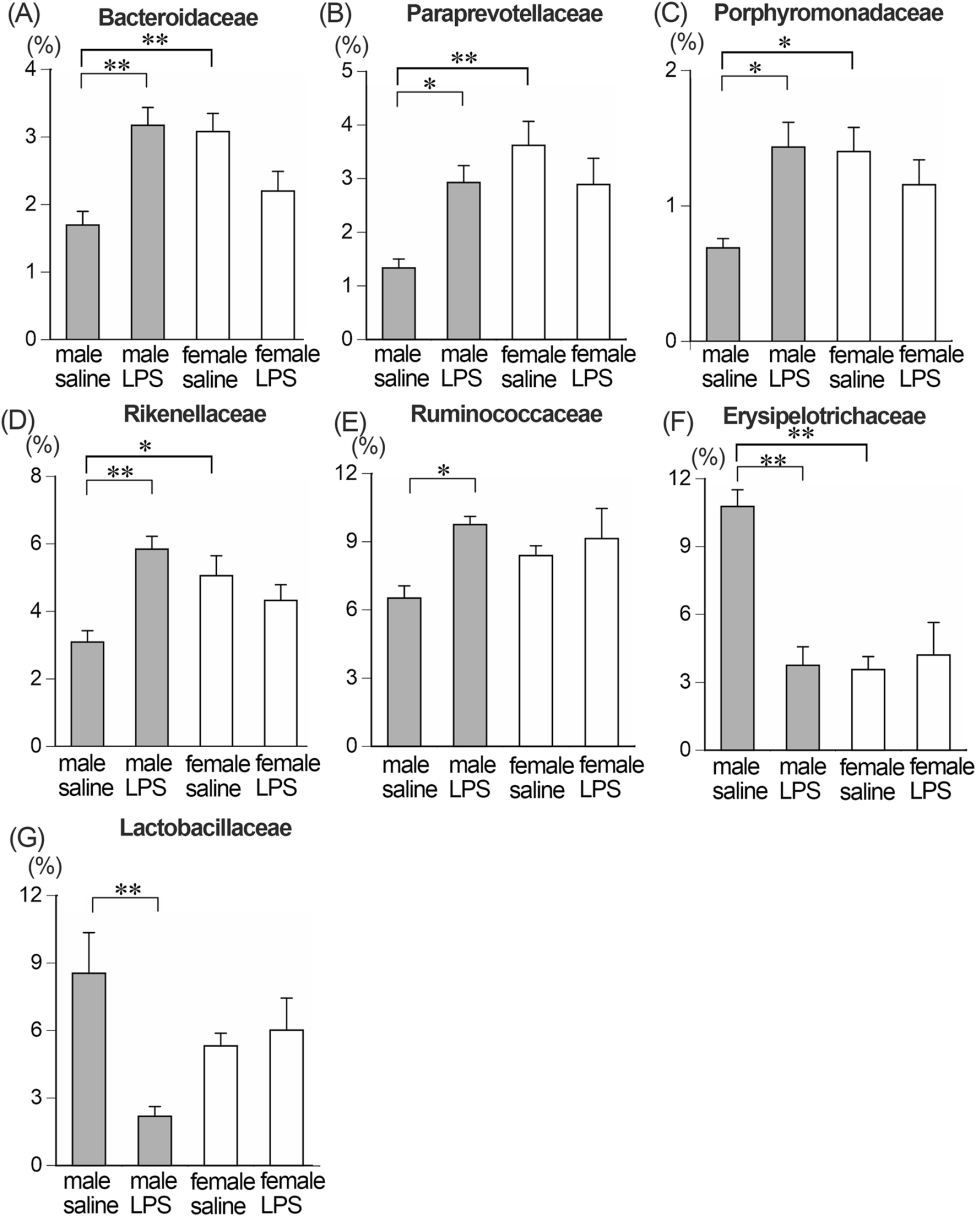
The relative abundance of gut microbiota at family level. Significant increases in the abundances of Bacteroidaceae (**A**), Paraprevotellaceae (k_Bacteria; p_Bacteroidetes; c_Bacteroidia; o_Bacteroidales; f_Paraprevotellaceae) (**B**), Porphyromonadaceae (**C**), Rikenellaceae (**D**), and Ruminococcaceae (**E**) and decreases in Erysipelotrichaceae (**F**) and Lactobacillaceae (**G**) in male LPS-treated compared to male saline-treated mice were detected. *p < 0.05, **p < 0.01. n = 5 for each group.

**Figure 4:**
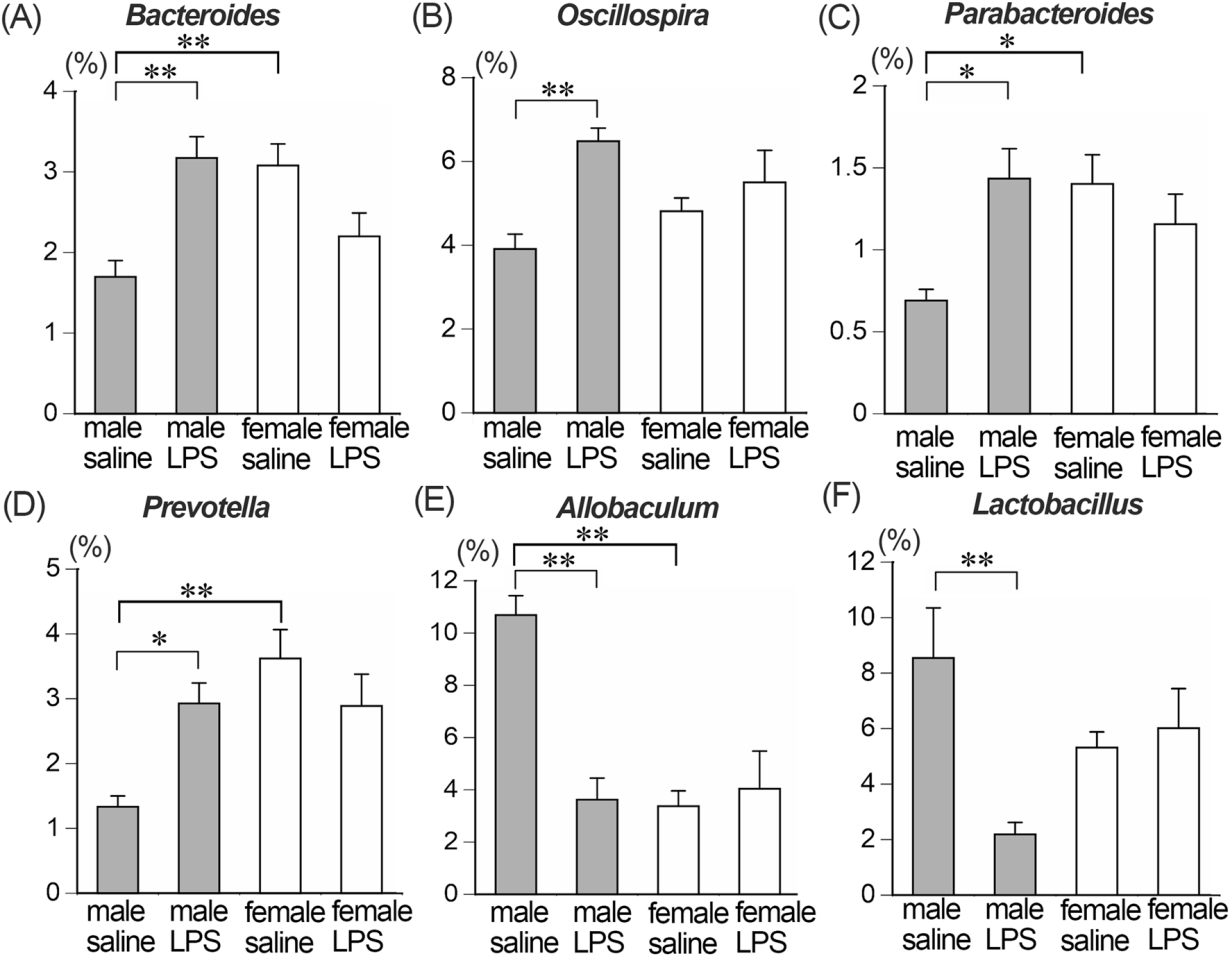
The relative abundance of gut microbiota at genus level. Significant increases in the abundances of *Bacteroides* (**A**), *Oscillospira* (**B**), *Parabacteroides* (**C**) and *Prevotella* (**D**) and decreases in *Allobaculum* (**E**) and *Lactobacillus* (**F**) in male LPS-treated compared to male saline-treated mice were detected. No significant changes in the abundance in female LPS-treated compared to female saline-treated mice were detected. *p < 0.05, **p < 0.01. n = 5 for each group.

At the family level of gut microbiota, five bacterial families significantly increased and two families significantly decreased after LPS treatment in male mice, whereas no bacterial family significantly changed in female mice amongst the 20 bacterial families analyzed (see “Materials and methods” section). The average relative abundances of Bacteroidaceae, Paraprevotellaceae (k_Bacteria; p_Bacteroidetes; c_Bacteroidia; o_Bacteroidales; f_Paraprevotellaceae), Porphyromonadaceae, Rikenellaceae and Ruminococcaceae increased and Erysipelotrichaceae and Lactobacillaceae decreased in male LPS-treated compared to male saline-treated mice (Fig. 3, Table 1). At the genus level, four bacterial genera increased and two decreased in male LPS-treated compared to male saline-treated mice, while no bacterial genera significantly changed in female LPS-treated mice amongst the 20 bacterial genera analyzed (see “Materials and methods” section). The abundance of *Bacteroides*, *Oscillospira*, *Parabacteroides* and *Prevotella* (k_Bacteria; p_Bacteroidetes; c_Bacteroidia; o_Bacteroidales; f_Paraprevotellaceae; g_*Prevotella*) significantly increased and *Allobaculum* and *Lactobacillus* significantly decreased in male LPS-treated mice (Fig. 4, Table 2).

**Table 1:** Perturbed bacteria at the family level.

Male			Female	
Bacterial names		Fold increase or decrease	Bacterial names	Fold increase or decrease
**Up**				
Bacteroidaceae		1.9		
Paraprevotellaceae*		2.2		
Porphyromonadaceae		2.1		
Rikenellaceae		1.9		
Ruminococcaceae		1.5		
**Down**				
Erysipelotrichaceae		0.3		
Lactobacillaceae		0.3		

*k_Bacteria; p_Bacteroidetes; c_Bacteroidia; o_Bacteroidales; f_Paraprevotellaceae.

**Table 2:** Perturbed bacteria at the genus level.

Male			Female	
Bacterial names		Fold increase or decrease	Bacterial names	Fold increase or decrease
**Up**				
*Bacteroides*		1.9		
*Oscillospira*		1.7		
*Parabacteroides*		2.1		
Prevotella*		2.2		
**Down**				
*Allobaculum*		0.3		
*Lactobacillus*		0.3		

*k_Bacteria; p_Bacteroidetes; c_Bacteroidia; o_Bacteroidales; f_Paraprevotellaceae; g_Prevotella.

### Systemic cytokines

**Figure 5:**
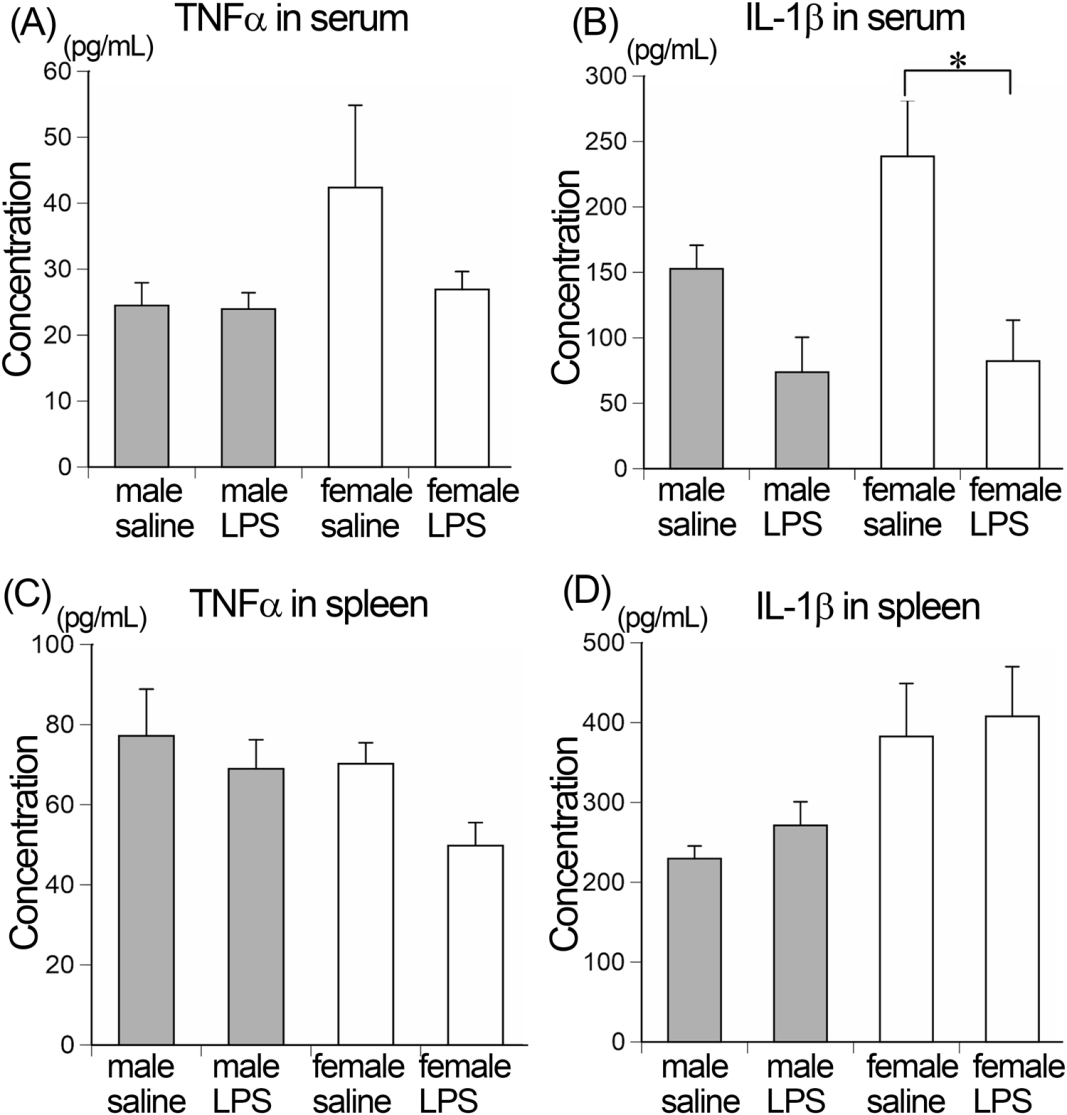
ELISA analysis. The concentrations of TNFα and IL-1β in the serum (**A** and **B**, respectively) and spleen extraction (**C** and **D**, respectively) of male saline, male LPS, female saline and female LPS group mice. There was no significant increase in LPS-treated male or female mice. n = 5 for each group.

To address the possibility that the chronic nasal inflammation caused systemic inflammation, we examined the expression levels of representative pro-inflammatory cytokines, TNFα and IL-1β, using serum and spleen extraction by ELISA analysis. The amounts of TNFα in serum were 24.5 ± 3.4, 24.0 ± 2.5, 42.4 ± 12.5 and 26.9 ± 2.7 pg/mL in male saline, male LPS, female saline and female LPS group mice, respectively, and did not differ between saline-treated control and LPS-treated male or female mice (Fig. 5A). The amounts of IL-1β in serum were 152.7 ± 18.0, 73.7 ± 26.7, 238.6 ± 42.8 and 82.2 ± 31.3 pg/mL in male saline, male LPS, female saline and female LPS group mice, respectively, and did not increase in LPS-treated male or female mice compared to saline-treated controls (Fig. 5B). The amounts of TNFα or IL-1β in the spleen extract did not differ between control and LPS-treated male or female mice (Fig. 5C, D).

## Discussion

This study is the first to draw a link between chronic nasal inflammation and perturbation of gut microbiota and that this is different in male and female mice. This may show that nasal inflammation affects the brain not only through the damage of the olfactory neural pathway but also by perturbing the gut-brain axis.

The Shannon index showed a lower heterogeneity of bacteria in male control mice compared to the other groups and the beta diversity in the Bray–Curtis dissimilarity showed a separation of plots in the male saline group compared to the other plots of different groups. Similarly, the Firmicutes/Bacteroidetes ratio significantly decreased only in male LPS-treated compared to male saline-treated mice but the ratio did not change in female mice and was similar to male LPS-treated mice. The abundances of higher numbers of bacteria changed after LPS treatment in male mice compared to female mice. In most cases, the abundance values for male LPS-treated mice approached those in female mice. These results suggest that male mice were more vulnerable to chronic nasal inflammation and tended to alter gut microbiota after LPS treatment. The fact that the heterogeneity (Shannon value) was lower in male mice than in female mice in the normal condition may explain the vulnerability in male mice. In addition, the results can be interpreted that the initial sex differences in the gut microbiota in saline-treated control mice were lost after chronic nasal inflammation, suggesting that the effects of nasal inflammation on the gut microbiota exceeded the intrinsic sex differences.

After long-term intranasal administration of LPS, the level of Bacteroidetes increased and Firmicutes decreased at the phylum level in male mice. At the genus level, *Bacteroides*, *Oscillospira, Parabacteroides* and *Prevotella* significantly increased, whereas *Allobaculum* and *Lactobacillus* decreased in LPS-treated male mice.

Higher Bacteroidetes and lower Firmicutes at the phylum level in the gut microbiota are known to be demonstrated in adult male mice after chronic restraint stress for 3 weeks^20^. Similarly, in human studies, Bacteroidetes, Proteobacteria and Actinobacteria significantly increase and Firmicutes significantly decrease in major depressive disorder patients compared to healthy controls^21^. Another study showed that severe stress is associated with lower relative levels of Firmicutes at the phylum level and higher *Bacteroides*, *Parabacteroides*, *Rhodococcus*, *Methanobrevibacter* and *Roseburia* but lower *Phascolarctobacterium* at the genus level in Belgian children^22^. In an older report, *Bacteroides* was shown to increase in astronauts during confinement in the Skylab test chamber with Skylab food, but not in those in the normal condition with Skylab food^23^. These reports suggest a relationship between chronic stress and increased Bacteroidetes at the phylum level and *Bacteroides* at the genus level.

In contrast, chronic stress induces the reduction of *Lactobacillus* at the genus level. The mice that have been exposed to chronic unexpected mild stress and those that received the microbiota from the stressed mice both show high levels of anxiety- and depressive-like behavior and change in the composition of gut microbiota. In these mice, *Lactobacillus* was lower in abundance, whereas *Akkermansia* was higher^24^. In another study, the genus *Lactobacillus* was reduced in the male mice exposed to the stressor^25^. Also, *Lactobacillus* as well as *Bifidobacterium* has been shown to be reduced in cosmonauts after space missions^26^. Since *Lactobacillus* has been shown to decrease in response to a psychological challenge, it has been used as a probiotic in humans and experimental animals to improve behavioral abnormalities^27^. For example, *Lactobacillus johnsonii* has been shown to attenuate stress-induced and intraperitoneal injection of LPS-induced anxiety-like behavior in male mice^28^; *Lactobacillus rhamnosus* reduced stress-induced anxiety- and depression-related behavior in male mice^29^; Supplementation of *Lactobacillus reuteri* prevented longstanding LPS-induced changes in anxiety-like behavior and stress-induced brain activation in male mice^30^. Together with these reports, our results demonstrated that male, but not female mice with chronic nasal inflammation are likely to suffer from chronic stress. The inflammatory state in the OB in LPS-treated mice may be transmitted via the olfactory neurocircuit to the higher brain regions, such as piriform cortex, hypothalamus, amygdala, hippocampus and prefrontal cortex, which are brain regions associated with the stress response. In fact, mental stress induces neuroinflammation in these brain regions, suggesting that brain responses to the peripheral inflammation are similar to those to the mental stress^31,32^. On the other hand, mice with chronic nasal inflammation may suffer from impaired sense of smell due to the loss of OSNs and the atrophy of the OB. This anosmia-like situation may cause the mice much stress, leading to changes in the gut and possibly the brain as a stress response. Examining the changes at the cellular and molecular levels in the higher brain regions could result in better understanding of the psychiatric condition of the mice and the mechanisms underlying chronic nasal inflammation-induced dysbiosis.

In the present study, the abundance of *Allobaculum* was also found to decrease after LPS treatment in male mice. The abundance of *Allobaculum* is reported to be negatively correlated to leptin concentrations in the serum^33^. Since leptin is a hormone that is secreted mainly by white adipose tissue and strongly suppresses food intake by acting at the hypothalamus, LPS-treated male mice in the present study may reduce their food intake due to the decreased abundance of *Allobaculum*. In fact, we observed an inhibition in the normal increase in body weight during the observation period in LPS-treated mice (Supplementary Figure S3), although the increase in body weight was also suppressed in female mice with no decrease in *Allobaculum*. The relationship between nasal inflammation, perturbation of gut microbiota, serum leptin level and amount of food intake is an avenue for future research.

Several studies have reported sex differences in the composition of gut microbiota under physiological conditions in humans and animals^34^. In this study, we also found sex differences in the gut microbiota in the normal condition (saline-treated mice). The ratios of Clostridiaceae, Christensenellaceae, Coriobacteriaceae and Erysipelotrichaceae were higher in males, while those of Bacteroidaceae, Dehalobacteriaceae, Paraprevotellaceae, Porphyromonadaceae and Rikenellaceae were higher in females at the family level (Supplementary Table S1). Similarly, at the genus level, the ratios of *Adlercreutzia*, *Allobaculum* and *Clostridium* were higher in male mice, whereas those of *Bacteroides, Dehalobacterium, Parabacteroides* and *Prevotella* were higher in female mice (Supplementary Table S2).

In addition, we found sex-dependent differences in the pattern of perturbation of gut microbiota after chronic nasal inflammation. A greater number of bacteria were perturbed by the nasal inflammation in male mice compared to female mice. However, it remains unclear whether chronic nasal inflammation causes the sex-dependent differences in the perturbation of gut microbiota or whether the initial differences in the gut microbiota may contribute to the different responses to nasal inflammation. Further experiments using male and female mice that display the same initial microbiota would give us more information on the sex-dependent differences in the response. In addition, it may be another limitation of this study that feces from male and female mice cannot be normalized since they should be housed separately. Taking this into consideration, accumulating data on the sex differences in gut microbiota may bring new findings related to the gut-brain interaction and provide an explanation of sex differences in the incidence of psychiatric disorders.

## Materials and methods

### Animals

Male and female C57BL/6J JmsSlc mice (Sankyo lab, Tokyo, Japan) were purchased at 6 weeks, acclimated for 2 weeks at our animal facility and entered the study at 8 weeks. Mice were consistently housed in groups separated by gender. The intranasal administration was performed according to our previous study^6–8^. Briefly, the mice were anesthetized with isoflurane and 10-µL physiologic saline (saline-treated mice) or LPS from *Escherichia coli* O55:B5 (1 mg/mL; Sigma) (LPS-treated mice) was administered to bilateral nostrils three times per week for 9 weeks. All protocols were approved by, and all methods were performed in accordance with the guidelines of the Institutional Animal Care and Use Committee of the Kyorin University Faculty of Health Sciences (Protocol ‘I17-09-02’). In addition, this study was carried out in compliance with the ARRIVE guidelines.

### Experimental procedure

**Figure 6:**
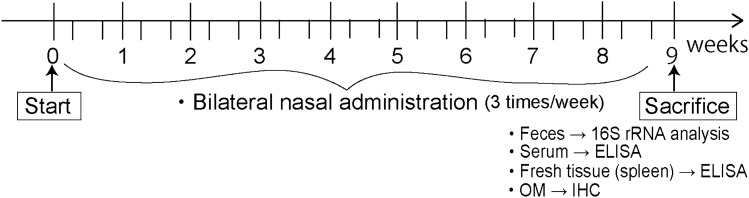
Experimental protocol. Mice received repeated bilateral intranasal administration of LPS or saline 3 times per week and were sacrificed at 9 weeks after the first administration. Feces, blood, fresh tissue of spleen were obtained for 16S rRNA and ELISA analyses. Olfactory mucosa (OM) was used for histological analysis.

All experiments were performed following the schedule in Fig. 6. Mice with bilateral nasal administration of saline or LPS (3 times/week) were sacrificed at 9 weeks after the first administration and 2 days after the last administration. At that time, mice were deeply anesthetized with ketamine and xylazine, whole blood was taken from the right ventricle and a piece of spleen was obtained and fresh frozen for enzyme-linked immunosorbent assay (ELISA) analysis. The feces from the cecum was obtained and fresh frozen by liquid nitrogen for 16S rRNA analysis. Other groups of mice were transcardially perfused with phosphate buffered saline followed by 4% paraformaldehyde after whole blood was taken from the right ventricle for histological analyses.

### Histological analysis

Frozen sections of OM were used for histological analysis. Briefly, post-fixation with the same fixative at 4 °C overnight, the rostral half of the calvaria and the nasal bone were decalcified by 2 × K-CX (Falma, Tokyo, Japan) for 2.5 h at room temperature. The OM was cryoprotected with 20% sucrose in PBS (wt/vol) at room temperature overnight, embedded in OCT compound (Sakura Finetek USA Inc., Torrance, CA), and maintained at − 80 °C until use.

Olfactory mucosa was coronally cut on a cryostat into 20 μm slices and was rehydrated with TBST (10 mmol/L Tris-HCl [pH 7.4] and 100 mmol/L NaCl with 0.1% Tween 20), blocked with blocking buffer (5% normal donkey serum [vol/vol] in TBST) at room temperature for 1 h. It was then incubated with primary antibodies diluted in blocking buffer overnight. The antibodies used in the present study were rat anti-F4/80- (1:200, Abcam, Cambridge, UK), rat anti-Ly-6G- (1:200, AdipoGen, Liestal, Switzerland), rabbit anti-CD3e- (1:150, Thermo Fisher Scientific, Waltham, MA), rat anti-CD45R (1:200, Abcam), goat anti-olfactory marker protein- (1:1000, Wako, Osaka, Japan), rabbit anti-GAP43- (1:1000, Novus Biologicals, Littleton, CO), rabbit anti-CD11b- (1:500, Abcam) and goat anti-IL-1β (1:200, R&D Systems, Minneapolis, MN) antibodies. Sections were incubated with host-matched secondary antibodies: Alexa Fluor 568- or 488-conjugated donkey anti-species IgGs (1:300, Thermo Fisher Scientific). Nuclei were counterstained with DAPI. The sections were coverslipped with fluorescence mounting medium (Dako Agilent, Santa Clara, CA) and imaged using a fluorescence microscope with structured illumination (BZ-X710; Keyence, Osaka, Japan).

### 16S metagenomic analysis

Genomic DNA was extracted from fecal samples and purified using Genomic DNA from stool samples (Macherey-Nagel GmbH & Co. KG, Germany). Each DNA specimen from feces was amplified using the Ion 16S Metagenomics Kit (Thermo Fisher Scientific, Bremen, Germany) as described in the previous study^35^. The amplicons were purified and prepared for the sequencing library by using the Ion Plus Fragment Library Kit (Thermo Fisher Scientific) and the Ion Personal Genome Machine (PGM) Hi-Q sequencing kit following the protocol of the kit. The sequencing runs were performed on the Ion PGM platform (Thermo). Unmapped bam files were converted to a fastq format using Torrent server (Thermo) by File Exporter v5.0.3.1.

Bioinformatic analysis of bacterial 16S rRNA amplicon data was conducted using the QIIME v1.9.1 software pipeline. Sequences were aligned and clustered into operational taxonomic units (OTU) based on the de novo OTU picking algorithm using the QIIME implementation of UCLUST with Greengenes database. OTUs with 97% similarity level were selected for taxonomical assignment and employed for the alpha diversity, richness (Chao 1) and evenness (Shannon) analysis. Bray–Curtis index was calculated as beta diversity. The counts in per sample were 32,476, 43,435, 53,816, 57,830, 62,106, 63,217, 64,877, 66,416, 71,516, 76,788, 79,871, 128,837, 136,371, 151,462, 159,118, 166,430, 169,062, 309,313, 347,304, and 660,519 and the depth of coverage was determined to be 32,476, the minimum value. Among sixty-eight families and one hundred and eighteen genera of bacteria found by the machine, 45 families and 46 genera were identified. We further analyzed 20 families and 20 genera that had more than 0.1% of existence in any one group (male saline, male LPS, female saline or female LPS).

### ELISA assay

Blood samples were stored at 4 °C overnight and centrifuged at 3500 rpm at 4 °C for 20 min. The supernatant serum was collected and stored at − 80 °C until use. The pieces of spleen were homogenized in 20 times volume of Tissue Protein Extraction Reagent (T-PER; Thermo Fisher Scientific, Waltham, MA) containing Halt Protease Inhibitor Cocktail, EDTA-free (Thermo Fisher Scientific) and centrifuged at 15,000 rpm at 4 °C for 5 min. The supernatant extract was collected and stored at − 80 °C until use.

The levels of TNFα and IL-1β in the serum and spleen extraction were determined by ELISA using DuoSet ELISA (R&D Systems, Minneapolis, MN) according to the manufacturer’s instructions. All samples were measured in duplicate. Values were compared between male saline, male LPS, female saline and female LPS group mice (n = 5).

### Statistical analysis

Microbiota and cytokine levels were compared statistically by 2-way analysis of variance, followed by Tukey’s HSD post hoc tests for multiple comparisons using Statistica software (Dell Software, Round Rock, TX). A *p* < 0.05 represented a significant difference. Values are reported as means ± SEMs.

## Supplementary Information

Supplementary Information.
