# Supplementary material for: Sex-dependent differences in the gut microbiota following chronic nasal inflammation in adult mice
Source: Sci Rep. 2021 Feb 25;11:4640. doi: 10.1038/s41598-021-83896-5 (PMC7907122; doi:10.1038/s41598-021-83896-5)
Supplement: Supplementary file 1 — Supplementary Information. [file 41598_2021_83896_MOESM1_ESM.docx]

**Sex-dependent differences in the gut microbiota following chronic nasal inflammation in adult mice**

Yuko Mishima^1^, Takako Osaki^2^, Atsuyoshi Shimada^3^, Shigeru Kamiya^2^, and Sanae Hasegawa-Ishii^3*^

**Affiliation**

^1^ Department of Immunology, Faculty of Health Sciences, Kyorin University, 5-4-1 Shimorenjaku, Mitaka, Tokyo, 181-8612, Japan

^2^ Department of Infectious Diseases, Kyorin University School of Medicine, 6-20-2 Shinkawa, Mitaka, Tokyo, 181-8611, Japan

^3^ Pathology Research Team, Faculty of Health Sciences, Kyorin University, 5-4-1 Shimorenjaku, Mitaka, Tokyo, 181-8612, Japan

**Contact information**

*Sanae Hasegawa-Ishii, sanae_ishii@ks.kyorin-u.ac.jp


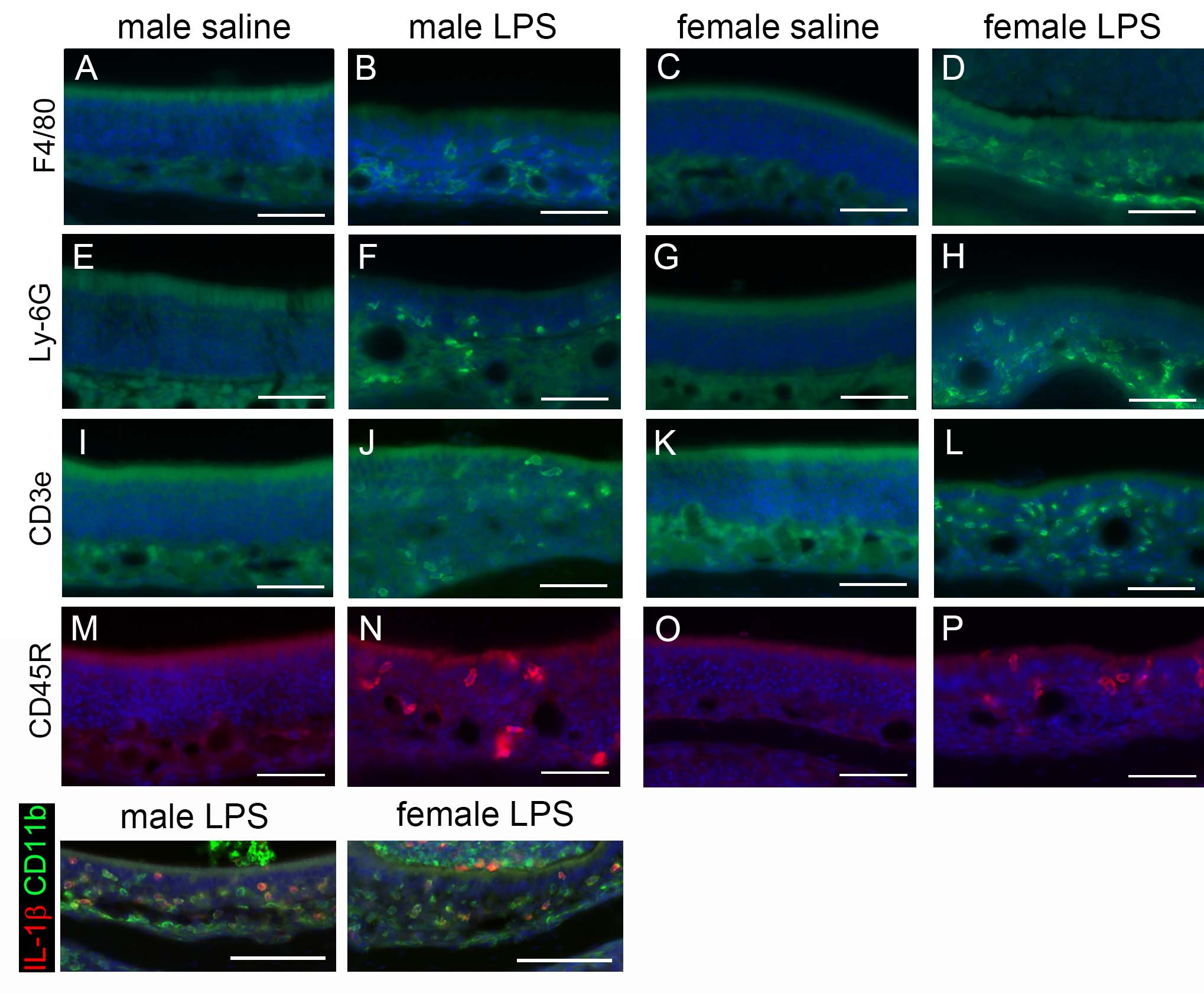
Supplementary Figure S1: Infiltration of inflammatory cells into the olfactory mucosa. (A-P) Almost no F4/80-positive (A, C), Ly-6G-positive (E, G), CD3e-positive (I, K) or CD45R-positive (M, O) cells are observed in the saline-treated olfactory mucosa in male (A, E, I and M) or female mice (C, G, K and O). F4/80-positive (B, D), Ly-6G-positive (F, H), CD3e-positive (J, L) and CD45R-positive (N, P) cells locally infiltrate the olfactory mucosa in the LPS-treated male (B, F, J and N) and female mice (D, H, L and P). (Q and R) IL-1β-positive cells (red) are observed in some regions of the LPS-treated olfactory mucosa, most of which are immunopositive for CD11b (green) in LPS-treated male (Q) and female (R) mice. Scale bars, 100 μm.


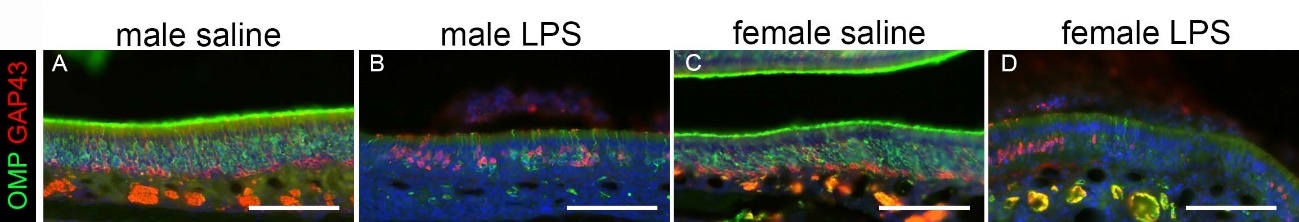
Supplementary Figure S2: Loss of olfactory sensory neurons. Immature olfactory sensory neurons (GAP43-positive, red) are located at the bottom of the olfactory epithelium underneath mature olfactory sensory neurons (OMP-positive, green) in saline-treated male and female mice (A, C). Repeated LPS administration causes loss of immature and mature olfactory sensory neurons at a similar level in male and female mice (B, D). Scale bars, 100 μm.


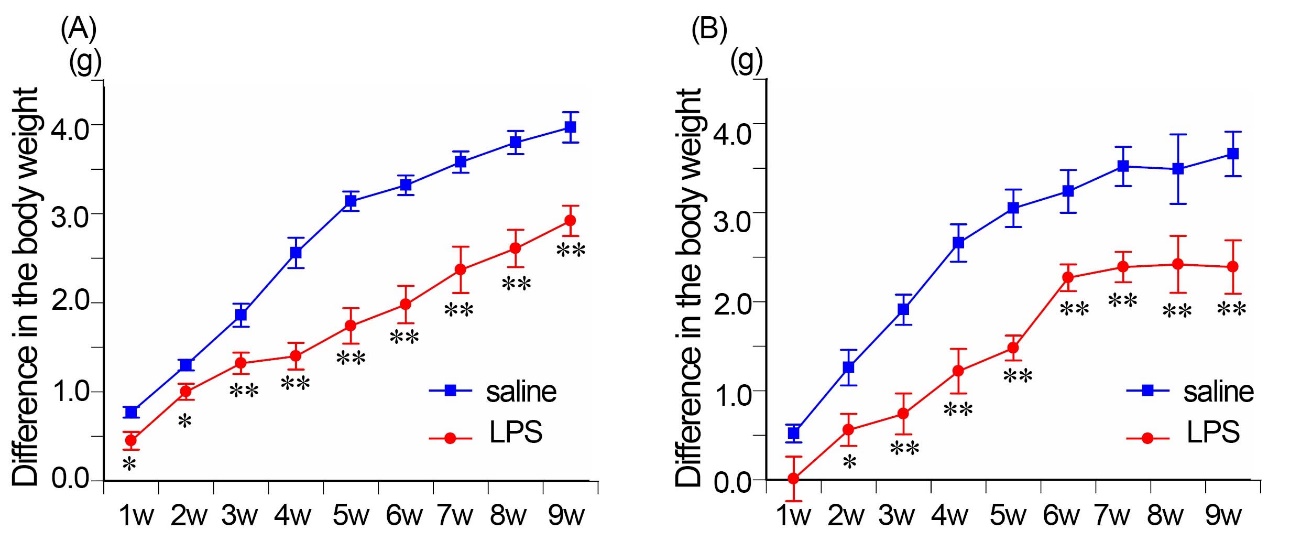


Supplementary Figure S3: Increase in the body weight. The increase in the body weight compared to the beginning is lower in LPS-treated male and female mice during the observation period. * p < 0.05, ** p < 0.01.

|  | Bacterial names | Fold increase or decrease |
| --- | --- | --- |
| **M>F** | Clostridiaceae Christensenellaceae  Coriobacteriaceae  Erysipelotrichaceae | 2.6  2.4  1.6  3.0 |
| **M<F** | Bacteroidaceae Dehalobacteriaceae Paraprevotellaceae*  Porphyromonadaceae  Rikenellaceae | 0.6  0.6  0.4  0.5  0.6 |

Supplementary Table S1. Difference in the ratio of gut microbiota between male and female mice at the family level.

*k__Bacteria;p__Bacteroidetes;c__Bacteroidia;o__Bacteroidales;f__Paraprevotellaceae

|  | Bacterial names | Fold increase or decrease |
| --- | --- | --- |
| **M>F** | *Adlercreutzia*  *Allobaculum*  *Clostridium* | 1.7  3.2  2.9 |
| **M<F** | *Bacteroides*  *Dehalobacterium*  *Parabacteroides*  *Prevotella** | 0.6  0.6  0.5  0.4 |

Supplementary Table S2. Difference in the ratio of gut microbiota between male and female mice at the genus level.

*k__Bacteria;p__Bacteroidetes;c__Bacteroidia;o__Bacteroidales;f__Paraprevotellaceae;g__*Prevotella*
